# Complications of transoral endoscopic thyroidectomy vestibular approach (TOETVA)

**DOI:** 10.1590/0100-6991e-20202557

**Published:** 2021-01-13

**Authors:** GILBERTO MENDES MENDERICO, ABRAHÃO LOTHAR WEISSENBERG, CLARA MARINHO BORBA, GIOVANNA MORALES SALLANI, JANAÍNA DE OLIVEIRA POY

**Affiliations:** 1 - Centro Universitário Lusíada, Disciplina de Clínica Cirúrgica do Curso de Medicina - Santos - São Paulo - Brasil; 2 - Colégio Brasileiro de Cirurgiões, Membro Adjunto - São Paulo - SP - Brasil; 3 - Centro Universitário Lusíada, Curso de Medicina - Santos - SP - Brasil

**Keywords:** Postoperative Complications, Thyroidectomy, Natural Orifice Endoscopic Surgery, Tireoidectomia, Complicações Pós-Operatórias, Cirurgia Endoscópica por Orifício Natural

## Abstract

The thyroidectomy is the most frequently executed procedure in head and neck surgery. Since its first description by Kocher, the transverse cervical incision has been the main access to the thyroid site, as it provides broad exposure of the central neck compartment. Despite the meticulous suture of the incision, the development of a scar with variable dimensions is unavoidable and, hence, some patients might not agree to the approach, due to this consequence. The transoral endoscopic thyroidectomy vestibular access (TOETVA) gains importance as an alternative to the traditional surgery, since it avoids the formation of visible scars. The objective of this study is to develop a systematic review on the currently available literature to evaluate possible complications related to the TOETVA. The systematic review was based on the databases of Medline, Cochrane library, Embase and Scielo/Lilacs, resulting in the selection of six studies, which were compared in regard of the type of study duration of the study and identified complications. Our study showed that TOETVA is related to complications similar to the ones identified in the conventional approach, such as hematoma, seroma, recurrent laryngeal nerve injury, hypoparathyroidism, surgical site infection. The TOETVA was associated to a higher risk of thermic injury of the skin and mentual nerve paresthesia. Moreover, it was possible to conclude that TOETVA is a safe procedure for well selected patients, with favorable conditions and concerned about the aesthetic outcome. The risk of complications of the procedure should always be explained to those patients.

## RESUMO

A tireoidectomia é o procedimento cirúrgico mais frequentemente realizado na cirurgia de cabeça e pescoço. Desde sua descrição por Kocher, a incisão cervical transversa constitui o principal acesso à loja tireoideana e permite ampla exposição à região central do pescoço. Apesar do fechamento meticuloso da incisão, uma cicatriz de dimensões variáveis é inevitável, e certos pacientes podem discordar de tal abordagem. A tireoidectomia vestibular endoscópica transoral (TOETVA) ganha importância como alternativa à cirurgia tradicional, pois evita a formação de cicatrizes visíveis. O objetivo de estudo é realizar uma revisão sistemática da literatura das possíveis complicações da TOETVA. Foi realizada revisão sistemática da literatura nas bases de dados Medline, The Cochrane Library, Embase e SciElo/Lilacs, sendo selecionados seis artigos e tabulados os dados de tipo de estudo, período do estudo e complicações apresentadas. O estudo mostra que a TOETVA apresenta complicações semelhantes à técnica convencional, como hematoma, seroma, lesão de nervo laríngeo recorrente, hipoparatireoidismo, além de infecção de sítio cirúrgico, com maior risco de lesão térmica da pele e parestesia por lesão do nervo mentual. Em adição, foi possível concluir que a TOETVA é uma técnica segura para pacientes bem selecionados, com condições favoráveis e com especial preocupação com resultados estéticos, devendo sempre ser orientados sobre possíveis complicações.

## INTRODUCTION

Thyroid gland surgery is the most common surgical procedure performed on the head and neck area^1^ ^,^ ^2^. Currently, the most widely used technique for thyroidectomy is similar to that described by Kocher in the late 1880s, with minor modifications^2^ ^-^ ^3^. Since its description, the transverse cervical incision constitutes the main access to the thyroid site in interventions on the thyroid and parathyroid glands due to the wide, central neck region exposure^3^. Despite the meticulous closure of the incision, scarring of varying degrees is inevitable, and certain patients may disagree with such an approach^3^. The increasing incidence of thyroid diseases, as well as younger patients at the time of diagnosis, the predominance of female patients, and society’s emphasis on the physical appearance of the human being, generated great search for the development of aesthetically favorable, alternative approaches^3^. 

Endoscopic neck surgery was first described by Gagner in 1996, with the minimally invasive techniques of thyroidectomy surgery developed over the past two decades, which spares the cervical region from a scar^4^. Such techniques include endoscopic or robotic incisions: mammary, axillary-mammary, axillary, and retroauricular^5^. However, all the techniques previously described lead to visible scars, sometimes larger or more prominent than those generated by the conventional technique^2^.

The increasing development of natural orifice transluminal endoscopic surgery (NOTES) has allowed the transoral thyroidectomy technique to gain prominence, as it completely prevents the formation of visible skin scars^6^. Two techniques have been described: the sublingual technique, related to severe tissue injury and a high rate of complications, and the transoral endoscopic thyroidectomy vestibular approach (TOETVA), which appears to be safer than the first one^6^ ^,^ ^7^.

The TOETVA access is in the vestibular area of the lower lip, through which the thyroid can be accessed with less manipulation of nearby nervous and vascular structures in comparison with the mammary, axillary-mammary, retroauricular, and open approaches^8^. Due to the location of the incisions, the scars resulting from this operation are not visible, which is the most important characteristic for the patient who wishes to avoid this aesthetic complication^8^. Current indications for TOETVA include reluctance towards visible surgical scarring and personal history of pathological or hypertrophic scarring in patients with a small volume thyroid^9^. On the other hand, its contraindications include patients with a previous history of surgery or radiation in the head and neck region, intolerance to general anesthesia, active oral infection, poorly controlled hyperthyroidism, suspicion or evidence of extra-thyroid extension of malignant thyroid lesions, and evidence of recurrent laryngeal nerve palsy^9^. Lymph node metastasis in the central neck region is considered a relative contraindication and, to date, metastases to the lateral neck compartment are formal contraindications to the procedure^9^. However, in view of the advent of new treatment modalities, the presence of initial resistance and skepticism is common, generating discussions about safety and the real benefits for the patient, with the exception of the aesthetic component^10^ ^,^ ^11^. 

During the phases of development and adoption of a new technique, there is necessarily a lack of high quality evidence about the said method. A robust collection of information on the implementation, results, and conditions are necessary for any implementation. The present study aims to present a review of the relevant literature^7^ ^-^ ^11^.

## OBJECTIVE

 The objective of the study is to carry out a systematic review of the literature to list the possible complications of transoral endoscopic thyroidectomy vestibular approach (TOETVA).

## METHODS

### Ethical aspects

This study was not submitted to the Ethics Council of the Institution involved because it is a systematic review, studying only already published articles. 

### Search strategy, inclusion and exclusion criteria

To conduct the systematic review, we searched the main databases Medline, Cochrane Library, Embase, SciElo, and Lilacs (Latin American and Caribbean Health Sciences). The search considered articles in Portuguese and English, published between 2015 and 2020, with a broad search strategy to avoid publication bias. We used combinations between the terms “thyroidectomy”, “toetva”, “transoral endoscopic vestibular approach thyroidectomy” and “complications”. 

We excluded articles in languages other than those mentioned above, or those that could not be fully recovered. we also excluded papers that did not contain sufficient data to evaluate the variables under study. 

We included studies on complications related to conventional thyroidectomy and transoral endoscopic thyroidectomy vestibular approach (TOETVA). We excluded articles that did not cover the subject. We used systematic review articles to discuss the results, not as part of them.

### Data analysis

We used The Microsoft Excel software (Microsoft Corp., Redmond, WA) to tabulate data, including type of study, country of origin, time taken to obtain the sample, type of operation performed, complication rates, and the association of the surgical approach with complications.

## RESULTS

**Figure 1:**
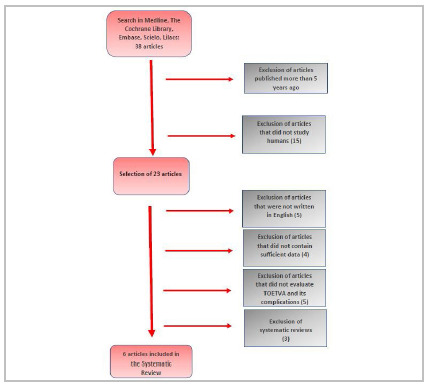
Flowchart of article selection.

We found 38 studies in the literature, of which we excluded 15 articles that did not study humans. From the remainder, we excluded those that did not appraise TOETVA and its complications, those that were not written in Portuguese or English, those that did not contain sufficient data, and systematic reviews, as shown in the flowchart of Figure 1. Thus, based on these criteria, we included six articles in the review, with results described in Tables 1 and 2 totaling 459 patients, 422 (91.9%) women aged between 16 and 85 years (mean 35.1).

**Table 1:** Articles analyzed in the literature on TOETVA.

Author	Period	Eligibility criteria	N	Study design	Thyroidectomy indication
Tesseroli MAS et al.^2^	12 months	Nodule < 4 cm in greater axis Gland volume < 35 cc Excluded: Previous cervical RT Previous cervical surgery Hyperthyroidism	9	Retrospective cohort	Thyroid Nodules Bethesda II (3) III (1) IV (2) V (3)
Anuwong A. et al.^7^	24 months	TPC Follicular lesion Thyroid lobe < 10 cm in greater axis; Graves’ disease Excluded: Previous cervical RT Previous cervical surgery TPC with lymph node metastasis or adjacent organ infiltration Diving goiter	425	Retrospective cohort	SUG / SMG / Follicular lesion (363) TPC (26) Graves’ Disease (33)
Kadem SG. et al.^13^	12 months	Female sex; Benign lesion < 4 cm in greater axis; Excluded: Male Obesity Short neck Previous RT Previous cervical surgery Thyroiditis Hyperthyroidism Malignant neoplasia	10	Case series	Uni / multinodular Goiter (10)
Bakkar S. et al.^14^	8 months	Age (30-45 years) Solitary nodule < 3.5 cm	5	Case series	Bethesda cytology classes: II (3) III (1) IV (1)
Wang Y. et al.^15^	3 months	Thyroid lobe up to 8 cm in greater axis Desire not to have scars Excluded: Suspected metastatic lymph nodes	10	Retrospective cohort	TPC (4) Follicular lesion (1) Suspected carcinoma (5)

N - population sample evaluated; SUG - simple uninodular goiter; SMG - simple multinodular goiter; TPC - thyroid papillary carcinoma; RT - radiotherapy.

**Table 2:** Type of thyroidectomy performed and complications.

Author	Type of surgery	Complications
Tesseroli MAS et al.^2^	TT: 7 PT: 2	Transient RLN injury: - Permanent RLN injury: 0 Transient hypoparathyroidism: - Permanent hypoparathyroidism: 0 Mental nerve palsy: - Hematoma: - Seroma: - Conversion to conventional technique: - Thermal injury: 1 SSI: -
Anuwong A. et al.^7^	TT (177) PT (245)	Transient RLN injury: 25 Permanent RLN injury: - Transient hypoparathyroidism: 43 Permanent hypoparathyroidism: 0 Mental nerve palsy: 33 Hematoma: 1 Seroma: 20 Conversion to conventional technique: 3 Thermal injury: - SSI: -
Kadem SG. et al.^13^	TT(0) PT(10)	Transient RLN injury: - Permanent RLN injury: - Transient hypoparathyroidism: - Permanent hypoparathyroidism: - Mental nerve palsy: 1 Hematoma: - Seroma: - Conversion to conventional technique: - Thermal injury: - SSI: -
Bakkar S. et al.^14^	TT (0) PT (5)	Permanent RLN injury: 0 Transient hypoparathyroidism: - Permanent hypoparathyroidism: - Mental nerve palsy: 0 Hematoma: 0 Seroma: - Conversion to conventional technique: 1 Thermal injury: 1 SSI: 0
Wang Y. et al.^15^	TT (1 ) PT (9)	Transient RLN injury: 0 Permanent RLN injury: 0 Transient hypoparathyroidism: 0 Permanent hypoparathyroidism: 0 Mental nerve palsy: - Hematoma: - Seroma: - Conversion to conventional technique: - Thermal injury: - SSI: -

PT - partial thyroidectomy; TT - total thyroidectomy; RLN - recurrent laryngeal nerve; SSI - surgical site infection.

## DISCUSSION

The surgical approach to the thyroid is the subject of many studies today, considering that surgery of the thyroid gland is the most common surgical procedure performed in the head and neck area, and that the new techniques under development raise doubts regarding possible complications^1^. 

The most significant complications of conventional thyroidectomy are hypoparathyroidism and dysphonia resulting from injury to the recurrent laryngeal nerve, in addition to bleeding and hematoma in the surgical bed that require immediate re-exploration, postoperative pain, and surgical site infection^12^.

 The 2017 study by Anuwong et al.^6^ covers TOETVA inclusion and exclusion criteria and allows to analyze whether the patient is eligible for the new technique. It appears that the inclusion criteria are configured by thyroid diameter less than 10 cm, benign tumor, follicular neoplasia, papillary microcarcinoma, Graves’ disease, and secondary, grade 1 goiter. The exclusion criteria are patient unfit for surgery and unable to tolerate anesthesia.

The 2018 study by Anuwong et al.^7^ compares complication rates between conventional thyroidectomy and TOETVA in 422 patients, including the 200 patients from the 2017 study of the same group, showing that TOETVA is not only associated with longer operations and less postoperative pain compared with conventional thyroidectomy, but presents results and complication rates similar to those of conventional thyroidectomy. In this study, they registered that no patient developed permanent hypoparathyroidism after the procedure, showing that the enlarged vision field provided by TOETVA can facilitate the identification of the parathyroid glands, thus decreasing the rate of this complication. In addition, patients in the TOETVA group had less postoperative pain based on the Visual Analogue pain scale. In contrast, TOETVA resulted in a greater risk of contamination of the surgical wound than conventional thyroidectomy, due to the location of the incision. However, there is still no consensus on the recommendation to use preoperative antibiotics for this surgical approach. 

The cohort study by Tesseroli et al.^2^ (2018), conducted in Brazil, analyzed nine patients who underwent TOETVA, with total thyroidectomy in eight cases. The complication reported was one thermal skin lesion in the chin region due to superficial dissection during the creation of the tunnel to pass the 10 mm trocar. There were no recurrent laryngeal nerve injuries or Permanent hypoparathyroidism. In addition, the use of TOETVA affected the patient’s concern with aesthetic issues. Moreover, when compared to other techniques, there are some advantages, such as a smaller dissection area and the possibility of access to the two lobes of the gland through the same incision. The authors point out that despite the pros, there are some limitations of TOETVA. For example, due to the size of the incision, it is sometimes necessary to fragment the gland to extract it, which could compromise the proper anatomopathological evaluation, such as margins, capsular invasion, and tumor size. 

Kadem et al.^13^ (2017) evaluated ten cases of TOETVA, without the need for conversion to conventional thyroidectomy. The only reported complications were one case of mild cervical emphysema that resolved completely within 24 hours and another case showing signs/symptoms of temporary mental nerve lesion, which were completely resolved within four weeks. The aesthetic results were highly satisfactory.

The study by Bakkar et al.^14^ (2017), on conventional and unconventional complications in five patients who underwent TOETVA, reported no cases of postoperative hemorrhage, hoarseness, mental nerve palsy, and surgical site infection. However, all patients developed subcutaneous emphysema with resolutions between 12 and 48 hours, and reported a sensation of uncomfortable traction throughout the surgery, which completely ceased within six months after the operation. In addition, one of the patients suffered thermal injury to the neck skin. 

To reduce the rate of complications related to TOETVA, new resources are being studied, as evidenced in the study by Wang et al.^15^ (2016), in which 10 patients were submitted to TOETVA with the use of neurophysiological monitoring of the recurrent laryngeal nerve (RLN). None of the 10 cases resulted in transient or permanent RLN paralysis. This demonstrates the promising benefit of the combination of resources.

Thus, studies show that patients strongly motivated to undergo a new surgical procedure adapted to their needs and desires must be adequately advised, particularly in relation to possible complications related to the new technique.

## CONCLUSION

The studies evaluated by this systematic review showed that TOETVA has complications similar to the conventional technique. Studies point out that TOETVA represents a higher risk of infection due to the surgical site and increased operative time. In addition, it was possible to conclude that TOETVA is a safe technique for well-selected patients, with favorable conditions, and with special concerns related to aesthetic results, and patients should always be advised about possible complications.
